# Ag-Embedded Laser-Induced Graphene as a Catalyst for the Oxygen Reduction Reaction in Alkaline Media

**DOI:** 10.3390/nano16191246

**Published:** 2026-10-02

**Authors:** Valentina Semenova, Sergey Pavlov, Alina Inozemtseva, Sergey Kislenko

**Affiliations:** 1 Joint Institute for High Temperatures of the Russian Academy of Sciences, Izhorskaya Str. 13 Bd. 2, 125412 Moscow, Russia; 2 N.N. Semenov Federal Research Center for Chemical Physics, Kosygina Str. 4, 119991 Moscow, Russia; 3 Moscow Center for Advanced Studies, Kulakova Str. 20, 123592 Moscow, Russia

**Keywords:** laser-induced graphene, silver nanoparticles, oxygen reduction reaction, solid-state synthesis, alkaline fuel cells, non-platinum catalyst, nitrogen-doped carbon, electrocatalysis

## Abstract

Oxygen reduction reaction (ORR) is a critical process in fuel cells, yet its slow kinetics necessitates efficient and cost-effective catalysts. Anion exchange membrane fuel cells (AEMFCs) offer a promising platform for ORR electrocatalysis, as the alkaline environment enables the use of non-precious metals and carbonaceous materials. Herein, we utilize a simple, solid-state, reagent-free, and one-step synthesis of silver nanoparticle-decorated laser-induced graphene (Ag@LIG) via direct CO_2_ laser writing on a polyimide film coated with an edible silver leaf. The laser treatment simultaneously converts the polymer into a porous three-dimensional graphene network and incorporates metallic silver nanoparticles (∼5–50 nm) into the carbon matrix. X-ray photoelectron spectroscopy confirms the metallic nature of the incorporated silver and reveals a higher nitrogen content in Ag@LIG than in pristine LIG, with pyrrolic nitrogen as the dominant configuration. Electrochemical characterization demonstrates that Ag@LIG exhibits enhanced ORR activity compared to pristine LIG, with a ∼30 mV positive shift in onset potential and a predominantly four-electron pathway over the entire potential range, with low peroxide formation. This work highlights the potential of the solid-state laser approach for producing Ag-carbon hybrid electrocatalysts with promising ORR performance for alkaline fuel cells.

## 1. Introduction

Oxygen reduction reaction (ORR) is a key cathodic reaction that largely dictates the energy conversion efficiency of fuel cells. However, the intrinsically sluggish kinetics of the multi-electron ORR process imposes a critical requirement for highly active electrocatalysts [1]. In the state-of-the-art proton exchange membrane fuel cells (PEMFCs), platinum-based materials remain the most efficient and practical catalysts [2,3]. Nevertheless, the prohibitive cost, limited natural reserves [4], and vulnerability to CO poisoning [5] severely hinder their large-scale commercialization. In response, considerable research efforts have been redirected toward anion exchange membrane fuel cells (AEMFCs) [6,7]. The alkaline environment is particularly advantageous because it enables the use of non-precious metals and carbonaceous materials, which exhibit high catalytic performance and stability compared to acidic systems [7]. Within this context, silver has emerged as a compelling candidate, offering a balanced trade-off between catalytic activity and economic viability [8,9,10]. Nevertheless, the practical application of Ag-based catalysts is often constrained by particle agglomeration and limited electrical conductivity, issues that can potentially be addressed through rational design of conductive carbon supports.

Various methods have been developed for the synthesis of carbon-supported metal catalysts, particularly Ag/C systems [10,11]. The conventional approach relies on commercially available carbon black as the support. To obtain the final Me/C catalyst, metal nanoparticles are deposited onto the carbon black surface from precursor salt solutions via chemical reduction or electrochemical deposition—a process that requires multiple steps, the use of reducing agents or applied potentials, and strict control over synthesis conditions. An alternative that has gained considerable attention is the use of laser-induced graphene (LIG) as a carbon support. LIG is produced via a one-step laser irradiation of polymer substrates, most commonly polyimide [12]. Upon exposure to CO_2_ laser radiation with a wavelength of 10.6 μm, the polymer undergoes rapid photothermal heating, resulting in localized carbonization and the formation of a three-dimensional porous sp^2^ carbon network. The resulting LIG exhibits high electrical conductivity, large specific surface area, and excellent mechanical flexibility, making it an attractive scaffold for catalytic applications [13,14,15]. Importantly, LIG synthesis is feasible under ambient air conditions, eliminating the need for inert atmospheres or vacuum systems—a significant practical advantage over conventional carbon black production. Furthermore, several recent studies have demonstrated the successful incorporation of various metal and metal oxide nanoparticles into LIG to form functional LIG-based composites [13,16], confirming the versatility of this platform for catalyst design.

To fabricate LIG-based catalysts, several strategies for incorporating nanoparticles into the LIG matrix have been developed. These can be broadly categorized into two-step and single-step approaches. The two-step method involves the initial synthesis of LIG, followed by impregnation with a precursor solution and a second laser treatment to form the LIG composite [17,18,19]. The single-step process simplifies fabrication by depositing a metal precursor-containing layer onto the polyimide film prior to a single laser exposure, which simultaneously carbonizes the polymer and reduces the metal ions to form nanoparticles [20,21]. Oxidation states of metal nanoparticles formed during laser processing correlate with the reduction potential. Metals with low reduction potentials, such as Fe, Co, Ni, and Mn, tend to form oxides, while noble metals like Ag, Cu, Au, Pt, and Pd yield metallic nanoparticles [22].

The described approaches share common limitations, including the risk of contamination from solvents and reducing agents, as well as the complexity of preparing and applying precursor solutions to the polymer substrate or pre-synthesized LIG. To address these limitations, a remarkably simple solid-state, reagent-free, and one-step synthesis method for noble metal–LIG composites has recently been introduced [23,24,25]. In this approach, a thin metal foil (or “metal leaf”) is placed directly onto the polyimide film prior to laser irradiation. The laser treatment simultaneously converts the polymer into LIG and incorporates the metal from the foil as nanoparticles, eliminating the need for solvents, precursors, or complex preparatory steps. While this technique has been successfully demonstrated for the fabrication of biosensors, its application in electrocatalysis—particularly for the oxygen reduction reaction—remains largely unexplored. In this study, we adopt this strategy to synthesize silver nanoparticle-decorated laser-induced graphene (Ag@LIG) and investigate its electrocatalytic performance for the ORR in an alkaline medium. We show that the incorporation of silver nanoparticles into LIG reduces the reaction overpotential and favors a four-electron reaction mechanism.

## 2. Experimental

### 2.1. Materials and Reagents

Kapton^®^ polyimide film (thickness 125 μm, DuPont, Wilmington, DE, USA) was used as the precursor for laser-induced graphene. Edible silver leaf (thickness 250 nm, food grade, E174) was obtained from a local commercial supplier and used as the silver source for the Ag@LIG composite. Potassium hydroxide (KOH, chemically pure grade) was purchased from RusKhim (Moscow, Russia). A 0.1 M KOH solution was prepared by dissolving the appropriate amount of KOH in deionized water. Nafion solution (5wt.%, DuPont, Wilmington, DE, USA) and isopropanol (chemically pure grade, RusKhim, Moscow, Russia) was used for catalyst ink preparation. Commercial Pt/C catalyst (40 wt.% Pt) was purchased from Prometheus R&D LLC (Rostov-on-Don, Russia). Oxygen (technical grade, 99.7%) and argon (high purity grade, 99.993%) were used for electrolyte saturation.

### 2.2. Ag@LIG and LIG Synthesis

Laser-induced graphene (LIG) and silver nanoparticle-decorated LIG (Ag@LIG) were synthesized via a one-step, reagent-free, solid-state laser direct writing approach. Commercial Kapton^®^ polyimide (PI) films were used as the carbon precursor. Prior to laser treatment, the PI films were cleaned with isopropanol to remove surface contaminants and dried in air.

For LIG preparation, the cleaned PI film was directly irradiated using a CO_2_ laser engraver (nominal laser power 40 W, Shanghai Sihao Machinery Equipment Co., Ltd., Shanghai, China) equipped with a movable optical head. The laser beam was moved line by line over the PI surface at a power setting of 10% of the maximum output and a scanning speed of 30 mm/s. These parameters yielded a porous, three-dimensional graphene network.

For Ag@LIG synthesis, a commercial edible silver leaf was placed onto the cleaned PI film. The same laser parameters and scanning pattern (10% power, 30 mm/s, line-by-line rastering) were then applied to the silver leaf-covered PI film. During laser irradiation, the photothermal conversion simultaneously carbonizes the underlying PI into a porous 3D graphene network and embeds silver nanoparticles (AgNPs) derived from the leaf into the LIG matrix. This solid-state process eliminates the need for metal precursor solutions, reducing experimental complexity and improving nanoparticle adhesion [23].

After synthesis, the LIG and Ag@LIG materials were carefully detached from the PI substrate by gentle scraping with a razor blade for electrochemical characterization and transmission electron microscopy (TEM) analysis. For physicochemical characterization, including scanning electron microscopy coupled with energy-dispersive X-ray spectroscopy (SEM-EDS), Raman spectroscopy, and X-ray photoelectron spectroscopy (XPS), the samples were investigated directly on the PI substrate without detachment.

### 2.3. Physico-Chemical and Morphological Characterizations

Raman spectroscopy was performed using an EnSpectr R532 spectrometer (Enhanced Spectrometry LLC, Chernogolovka, Russia) equipped with a 532 nm 30 mW laser.

Scanning electron microscopy (SEM) was performed using a Prisma E microscope (Thermo Scientific, Prague, Czech Republic) with a 5–10 kV acceleration voltage. Samples were preliminarily coated with 40 nm layer of gold by Q150R ES plus sputter coater (Quorum Technologies, London, UK). Secondary electron images were obtained under high vacuum conditions.

Transmission electron microscopy (TEM) analysis was conducted using a JEOL JEM-100CXII microscope (JEOL Ltd., Osaka, Japan) operated at an acceleration voltage of 100 kV. The Ag@LIG powder was ultrasonicated in isopropanol for 5–10 min. A drop of the suspension was deposited onto a 300-mesh copper grid coated with an amorphous carbon film and air-dried at room temperature prior to TEM examination.

X-ray photoelectron spectroscopy (XPS) measurements were carried out on a Thermo Fisher Scientific Escalab 250Xi spectrometer (Thermo Fisher Scientific, Waltham, MA, USA). High-resolution XPS spectra were processed using the Unifit 2014 software package. The spectral background was optimized using Tougaard functions [26] simultaneously with spectral fitting. The spectra were fitted by Gaussian/Lorentzian convolution functions. The quality of each fit was assessed by the Abbe criterion, with values close to unity indicating a good fit. For the Ag 3d and C 1s sp^2^ components, an asymmetric Doniach-Šunjić lineshape was employed to account for the asymmetry arising from the metallic nature of silver and the delocalized π-electron system of graphene, respectively. The asymmetry parameters were determined via free optimization. Quantification of the elemental composition and the relative fractions of different chemical states was performed by normalizing the peak areas obtained from the fits to the appropriate relative sensitivity factors (RSFs) [27].

### 2.4. Electrochemical Characterization

All electrochemical measurements were performed in a standard three-electrode cell using a 0.1 M KOH aqueous solution as the electrolyte. A platinum mesh and a Hg/HgO electrode (1 M KOH) served as the counter and reference electrodes, respectively. A Pine Research potentiostat (WaveDriver Model AFP2, Pine Research Instrumentation, Durham, CA, USA) coupled with a rotator (AFMSRCE, Pine Research) was used for all measurements. A rotating ring-disk electrode (RRDE) with a glassy carbon disk insert (5.0 mm diameter) and Pt ring (E6R1 ChangeDisk series, Pine Research) was employed as the working electrode substrate.

Prior to catalyst deposition, the glassy carbon disk was polished to a mirror finish with alumina slurries (down to 0.05 μm) and thoroughly rinsed with deionized water and isopropanol, and finally sonicated in deionized water for 5 min. Catalyst inks were prepared by dispersing the catalyst powder (24 mg of LIG, 24 mg of Ag@LIG, or 40 mg of commercial Pt/C (40 wt% Pt on Vulcan), which corresponds to 24 mg of carbon support and 16 mg of Pt) in a mixture of 1500 μL deionized water, 500 μL isopropanol, and 15 μL of 5 wt% Nafion solution, which was shown to be the optimal ink composition for Pt/C catalyst [28]. The suspension was homogenized using a mechanical homogenizer (10 min), followed by magnetic stirring (5 min) and ultrasonication (10 min) in two consecutive cycles. An aliquot of 12 μL of the ink was drop-cast onto the polished GC disk at 700 rpm and dried under rotation for 40 min to form a uniform catalyst film. This gives equal carbon loading of Pt/C catalyst and LIG of 0.73 mg/cm^2^, while for Ag@LIG it is lower (∼0.43–0.64 mg/cm^2^ according to EDS at.% of Ag in Ag@LIG) because we used the same powder mass as for LIG to avoid overestimating the Ag@LIG loading. All resulting loadings are summarized in Table 1.

Cyclic voltammetry (CV) was performed in Ar-saturated 0.1 M KOH at 5 mV s^−1^ over the potential range from −0.9 to +0.6 V vs. Hg/HgO. For ORR measurements, the electrolyte was saturated with O_2_, and CV was recorded over the potential range from −0.9 to +0.2 V vs. Hg/HgO. Subsequently, linear sweep voltammetry (LSV) was recorded using the RRDE tip at 5 mV s^−1^ from −0.9 to +0.2 V vs. Hg/HgO at 1500 rpm. The ring electrode was poised at +0.3 V vs. Hg/HgO to calculate the electron transfer number (*n*). All measurements were conducted at room temperature (25 ± 1 °C).

## 3. Results and Discussions

### 3.1. LIG and Ag@LIG Morphology and Composition

#### 3.1.1. Scanning and Transmission Electron Spectroscopies

**Figure 1:**
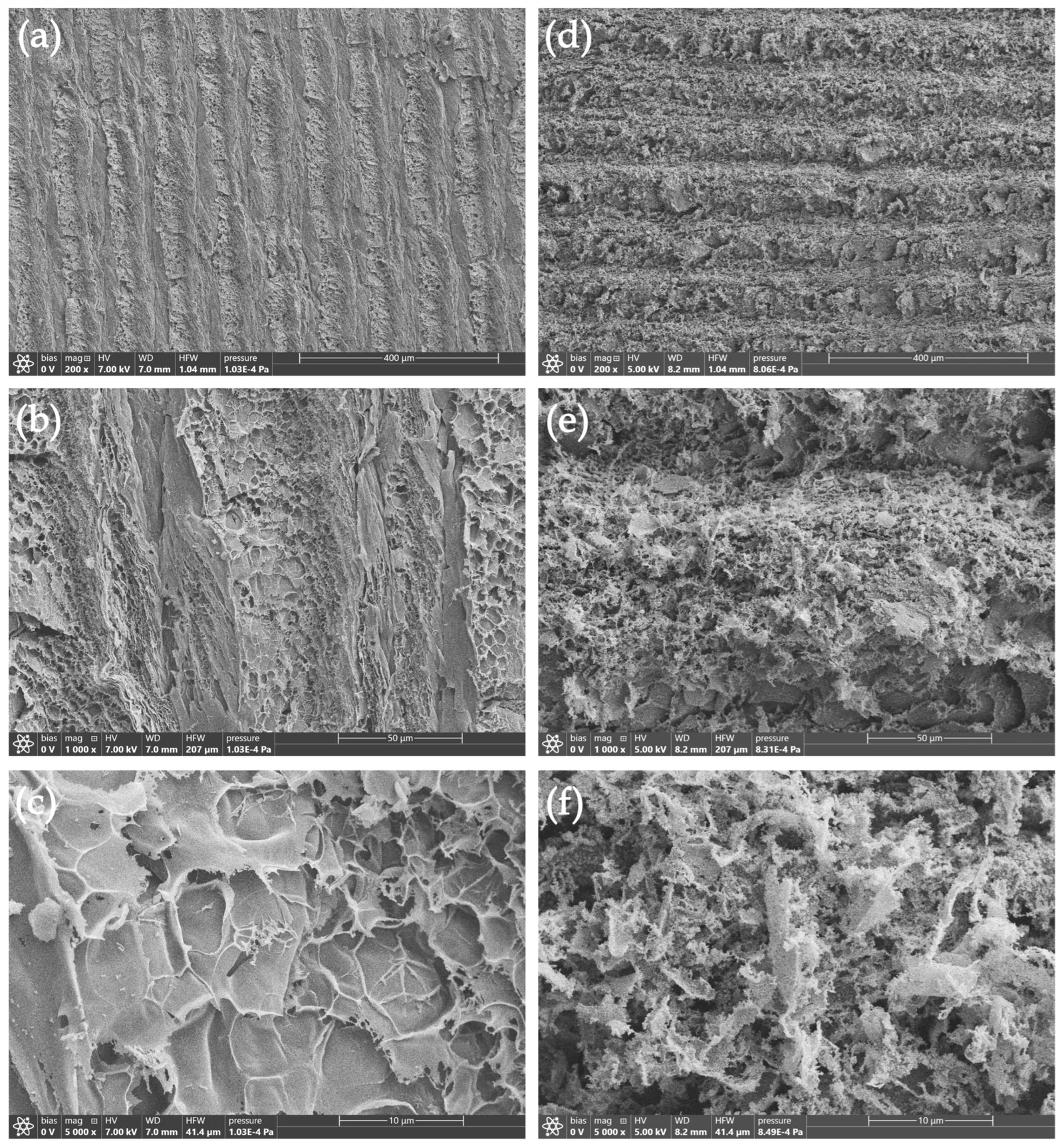
SEM images of LIG samples (**a**–**c**) and Ag@LIG samples (**d**–**f**) at three different magnification ratios.

The surface morphology of the as-prepared LIG and Ag@LIG samples was first examined by scanning electron microscopy (SEM) at various magnifications (Figure 1). At low magnification (Figure 1a,d, scale bar 400 μm), both samples exhibit distinct parallel line patterns with a line spacing of approximately 100 μm, which arise from the line-by-line scanning mode of the laser beam during the writing process and have been widely reported in the literature [12,14,29]. At intermediate magnification (Figure 1b,e, scale bar 50 μm), both LIG and Ag@LIG reveal a continuous porous network with interconnected flake-like structures.

At higher magnification (Figure 1c, scale bar 10 μm), the pristine LIG surface appears as plate-like morphology with abundant graphene nanosheets, folds, and pores. This structure is consistent with the formation of few-layer graphene sheets during the rapid photothermal decomposition and graphitization of the polyimide precursor [29,30]. The Ag@LIG sample retains this porous morphology, confirming that the presence of the silver leaf does not disrupt the laser-carbonization process. However, at high magnification (Figure 1f, scale bar 10 μm), a distinctly different morphology is observed: the surface appears more disordered and is characterized by a fibrous, nanofiber-like structure interspersed within the porous graphene network. Such morphological variations in LIG—from sheet-like to fibrous or foam-like structures—are known to be strongly dependent on the laser processing parameters, particularly laser power and scanning speed [29,30,31]. The observed transformation in Ag@LIG is likely attributable to the modified local thermal environment during laser irradiation caused by the presence of the silver leaf, which may alter the heat distribution and induce different carbonization pathways. Notably, no significant large-scale aggregates or continuous silver films are observed on the surface, indicating that the silver leaf is effectively disintegrated and incorporated into the graphene matrix during laser irradiation.

**Figure 2:**
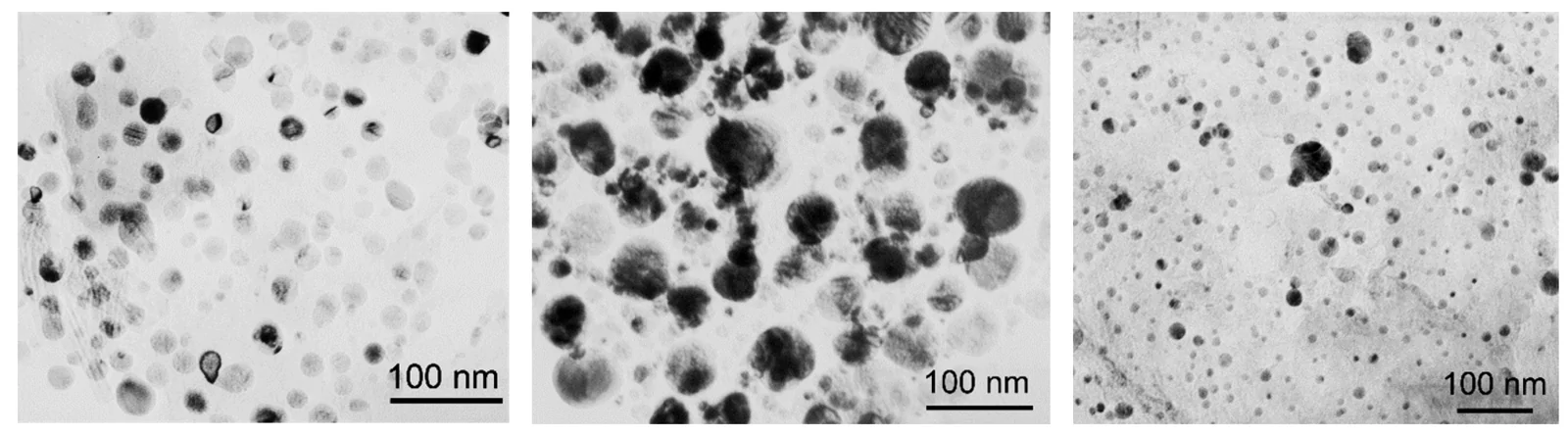
TEM images of Ag@LIG sample.

Transmission electron microscopy (TEM) was performed on the Ag@LIG sample to elucidate the size, morphology, and distribution of the embedded silver nanoparticles (Figure 2). The TEM images reveal the presence of Ag nanoparticles with predominantly spherical morphology, which are well embedded within the porous graphene matrix. The nanoparticles appear as dark, high-contrast features uniformly dispersed across the graphene sheets, indicating strong interfacial interaction between the AgNPs and the carbon support. Notably, the nanoparticles exhibit similar sizes within a given microscopic region, while the particle size varies significantly across different regions of the sample, ranging from approximately 5 to 50 nm. This spatial heterogeneity in particle size is attributed to the thermal gradient established across the laser-irradiated zone during the rapid photothermal conversion process.

#### 3.1.2. Raman Spectroscopy

**Figure 3:**
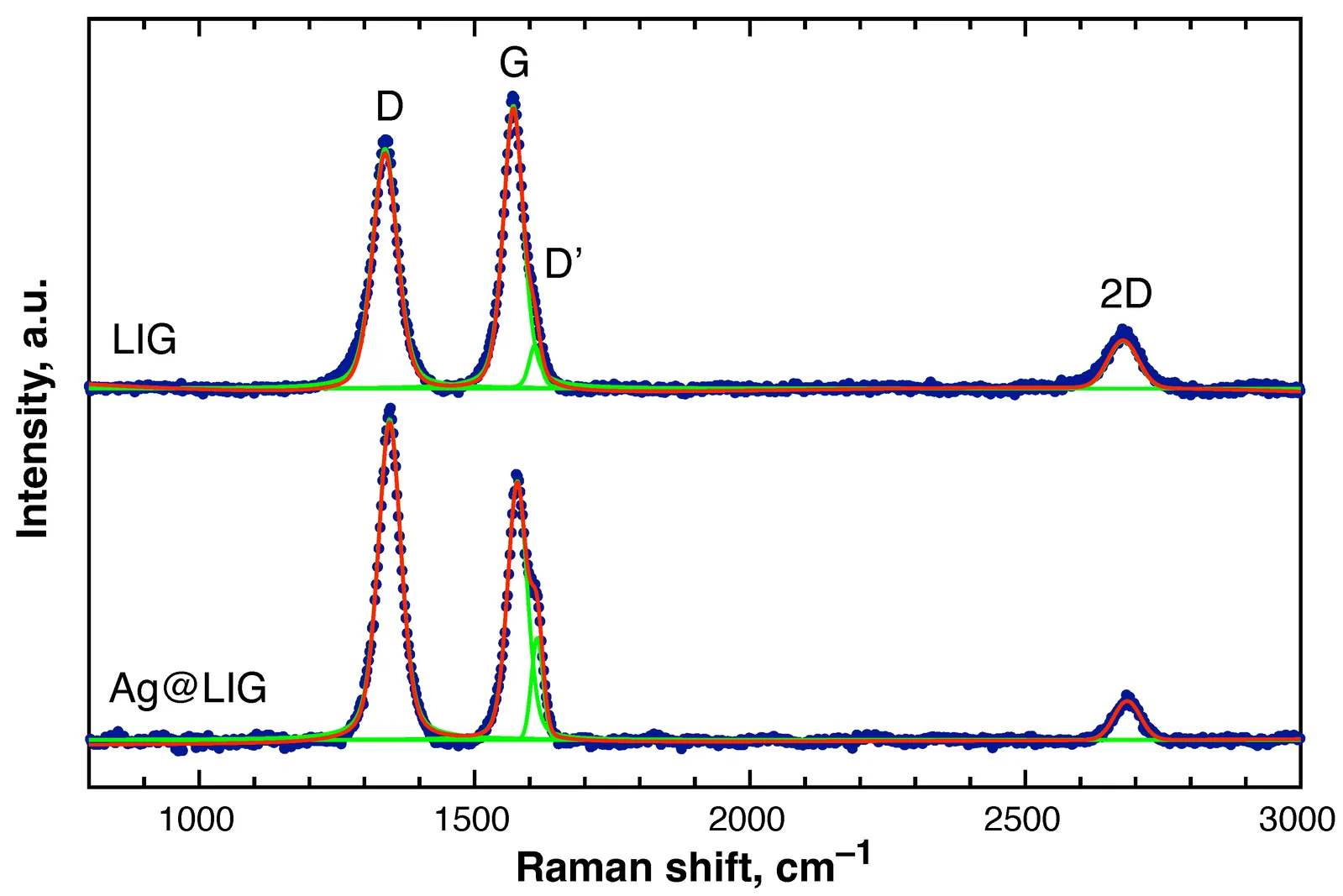
Raman spectra of the LIG and Ag@LIG samples. Blue dots: experimental spectra; green lines: individual components; red line: sum of the components.

Raman spectroscopy was employed to assess the structural quality of both LIG and Ag@LIG samples (Figure 3). The spectra display the characteristic D, G, $D^{\prime }$, and 2D bands typical of disordered graphitic carbon materials. The G band (∼1570 cm^−1^) originates from the in-plane stretching vibration of sp^2^ carbon pairs and is present in all graphitic materials [32,33]. The D band (∼1340 cm^−1^) arises from a double-resonance Raman process involving phonons near the K-point of the Brillouin zone and is activated by structural defects, such as vacancies, sp^3^-hybridized carbon, or edges [34,35,36]. The $D^{\prime }$ band (∼1610 cm^−1^) is also defect-activated and originates from an intravalley double-resonance process [32,37]; its relative intensity with respect to the D band ($I_{D}$/$I_{D^{\prime }}$) is particularly useful for distinguishing between different types of defects [38]. Finally, the 2D band (∼2680 cm^−1^) is the second-order overtone of the G band and does not require a defect to be activated. Its position, shape, and intensity ratio relative to the G band ($I_{2D}$/$I_{G}$) are widely used to assess the number of layers and the degree of disorder in graphene.

Upon incorporation of Ag nanoparticles, all Raman bands exhibit a blue shift of ∼8 cm^−1^ (see Table 2). A similar effect was previously observed for N-doped graphene samples and was attributed to a combined influence of electron doping by nitrogen atoms and compressive strain introduced by the five-membered pyrrole rings [39]. In addition to nitrogen doping, the presence of silver nanoparticles can induce charge transfer doping of graphene. The observed blue shift of both G and 2D bands is consistent with p-type doping of the graphene lattice by Ag [40], which further contributes to the overall shift. Therefore, the observed shift can be attributed to the combined effect of the increased nitrogen content and p-type doping induced by the Ag nanoparticles. The intensity ratio of the D to G band ($I_{D}$/$I_{G}$), commonly used as an indicator of structural disorder, increases from 0.86 in pristine LIG to 1.24 in Ag@LIG, indicating a significant rise in defect density [41,42].

The intensity ratio of the D’ to G band ($I_{D^{\prime }}$/$I_{G}$) increases markedly from 0.069 in pristine LIG to 0.21 in Ag@LIG. This significant enhancement of the D’ band, which is activated by intravalley scattering at defect sites, is consistent with the substantially higher nitrogen content in the Ag@LIG sample and is characteristic of nitrogen-induced defects, particularly those associated with pyridinic and pyrrolic nitrogen configurations [43]. More informatively, the intensity ratio of the D to $D^{\prime }$ band ($I_{D}$/$I_{D^{\prime }}$), which is sensitive to the nature of defects [38,44], decreases from ∼6 in pristine LIG to ∼3 in Ag@LIG. According to the literature, values of this ratio can be used to distinguish between different types of disorder: values around 7 are characteristic of vacancy-like defects, whereas values around 3.5 are associated with graphene edges or boundaries [38]. The observed decrease in $I_{D}$/$I_{D^{\prime }}$ therefore suggests a transition from vacancy-type defects in the pristine LIG to boundary-like defects upon silver incorporation. A similar evolution has been reported for nitrogen-doped graphene, where pyrrolic nitrogen dopants behave similarly to boundary defects [39], although the origin of this behavior was not discussed in detail.

Additionally, the presence of Ag nanoparticles may contribute to the observed spectral changes through surface-enhanced Raman scattering (SERS) effects. Ag nanoparticles are known to enhance Raman signals via localized surface plasmon resonance and charge transfer mechanisms [45]. However, the observed increase in the defect-related bands (D and $D^{\prime }$) and the change in the $I_{D}$/$I_{D^{\prime }}$ ratio are primarily attributed to structural modifications of the graphene lattice rather than to SERS alone. Furthermore, the $I_{2D}$/$I_{G}$ ratio decreases from 0.18 to 0.15, which is consistent with increased lattice disorder and electron doping effects [39,46,47]. Collectively, the Raman data indicate that the incorporation of Ag nanoparticles leads to a more disordered graphene structure and a change in the predominant type of defects, which may be influenced by the significantly higher nitrogen content in the composite.

#### 3.1.3. X-Ray Photoelectron Spectroscopy

High-resolution X-ray photoelectron spectroscopy (XPS) was employed for both qualitative and quantitative chemical analysis of the LIG and Ag@LIG samples.

**Figure 4:**
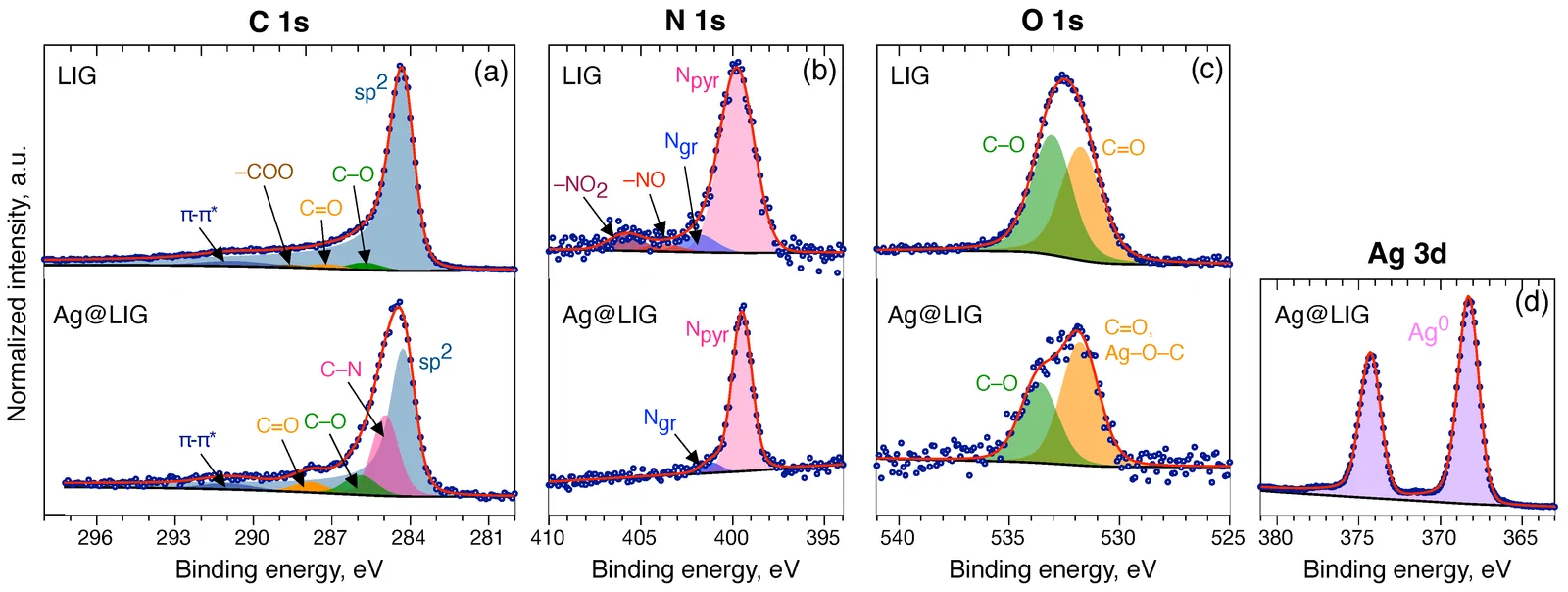
High-resolution XPS spectra of the pristine LIG and Ag@LIG samples: (**a**) C 1s, (**b**) N 1s, (**c**) O 1s, and (**d**) Ag 3d core-level regions. Blue dots: experimental spectra; colored regions: individual components; red line: sum of the components.

The Ag 3d spectrum of the Ag@LIG sample exhibits an asymmetric doublet, with the Ag ${3d}_{5/2}$ peak positioned at 368.2 eV (Figure 4d), corresponding to metallic silver [48]. The asymmetry of the Ag 3d peak, characteristic of metallic silver, was adequately described using the Doniach-Šunjić line shape, yielding an asymmetry parameter α of 0.049 from free optimization. This value is in good agreement with literature reports for metallic silver and silver clusters, where α values typically range from 0.00 to 0.10 [48]. Quantitative analysis demonstrates a substantial loading of Ag nanoparticles (24 at.%, Table 3), reflecting the successful incorporation of Ag into the LIG matrix.

The N 1s spectra of both samples are dominated by a single component at ∼399.5 eV, which is characteristic of pyrrolic nitrogen [49,50,51] (Figure 4b, Table 3 and Table 4). The predominance of pyrrolic nitrogen is consistent with previous reports on laser-assisted synthesis, where pyrrolic nitrogen is commonly the dominant configuration [45,52,53]. A minor component at ∼401.2 eV, corresponding to graphitic nitrogen [49,50], is also present in both samples. In the LIG sample, small additional components at higher binding energies (∼404–406 eV) are observed, suggesting the presence of a minor fraction of oxidized nitrogen species (e.g., –NO, –NO_2_) [54,55]; these components are absent in the Ag@LIG sample. The total nitrogen content is higher in Ag@LIG (∼11 at.%) than in pristine LIG (∼2 at.%), with pyrrolic nitrogen as the dominant configuration in both samples (Table 3 and Table 4). This increase may be related to the stabilization of nitrogen species through interaction with silver, as previously reported for nitrogen-doped carbon materials modified with metal nanoparticles [56]. However, the nitrogen content should be considered with caution, as it may not be stable under all conditions, and its exact role in the ORR performance requires further investigation.

The C 1s spectra of the LIG and Ag@LIG samples show a main peak for sp^2^-hybridized carbon at 284.2 eV (Figure 4a, Table 3 and Table 4). This peak in both samples was fitted using an asymmetric Doniach-Šunjić lineshape, as the delocalized π-electron system of graphene imparts a metallic character to the photoemission response [57]. The asymmetry parameter α obtained from free optimization was 0.17, which is in good agreement with the literature values reported for graphene and related graphitic materials (typically 0.05–0.20) [57]. Deconvolution of the C 1s spectra reveals the presence of oxygen-containing functional groups in addition to the main sp^2^ component. In the LIG sample, peaks corresponding to C–O (hydroxyl/ether, chemical shift CS = +1.49 eV [58]), C=O (carbonyl, CS = +2.95 eV [59]), and O–C=O (carboxylate, CS = +4.39 eV [58,60]) groups were identified (Figure 4a). A broad peak at 290–291 eV corresponds to a π–π* transition [61]. In the Ag@LIG sample, the relative intensities and binding energies of the oxygen-containing groups are modified. Notably, the main sp^2^ component is broadened, which may be attributed to the increased contribution from a C–N component [51,62] (CS = +0.77 eV), consistent with the higher nitrogen content in this sample (Table 3). The C–O and C=O components shift to higher binding energies (chemical shifts +1.7 eV and +3.6 eV, respectively) relative to the pristine LIG sample (Figure 4a, Table 4). This effect may arise from an increase in the work function [63,64] due to the higher abundance of nitrogen atoms, or from interactions of oxygen groups with Ag and the formation of interfacial Ag–O–C bonds [65].

The O 1s spectrum of the pristine LIG sample consists of two main components at ∼533.0 eV and ∼531.7 eV (Figure 4c, Table 3). The component at higher binding energy (∼533.0 eV) is typically assigned to C–O bonds (hydroxyl/ether groups [58]); the component at lower binding energy (∼531.7 eV) corresponds to C=O (carbonyl) groups [58]. In the Ag@LIG sample, the relative intensity of the component at ∼531.7 eV increases markedly, while the component at ∼533.6 eV correspondingly decreases (Figure 4c, Table 4). This redistribution of oxygen species is consistent with the formation of Ag–O–C bonds at the interface between the silver nanoparticles and the graphene oxide surface, as previously reported for metal–polymer systems [65]. The overall O/C ratio remains similar in both samples, indicating that the observed changes are due to chemical restructuring rather than a net change in oxygen content.

In summary, the XPS analysis reveals a substantial silver loading (24 at.%) in the Ag@LIG sample, along with a higher nitrogen content compared to pristine LIG. The observed shifts in the C 1s and O 1s spectra indicate a chemical interaction between silver nanoparticles and the graphene matrix, most likely through the formation of Ag–O–C interfacial bonds. These findings confirm that Ag interacts with the functional groups of LIG, resulting in a modified hybrid interface.

#### 3.1.4. Energy-Dispersive X-Ray Spectroscopy

The elemental composition of Ag@LIG was further assessed by energy-dispersive X-ray spectroscopy (EDS). The EDS analysis was performed on two distinct areas of the sample surface, yielding silver contents of approximately 7.3 at.% and 1.6 at.%, respectively (see Table 5). This spatial variation in Ag concentration is attributed to the inhomogeneous temperature distribution across the laser-irradiated zone, which arises from the line-by-line scanning pattern and the inherent thermal gradient during the photothermal conversion process. This observation is consistent with the broad nanoparticle size distribution (5–50 nm) observed by TEM (see Figure 2), which also reflects local temperature variations during synthesis.

**Figure 5:**
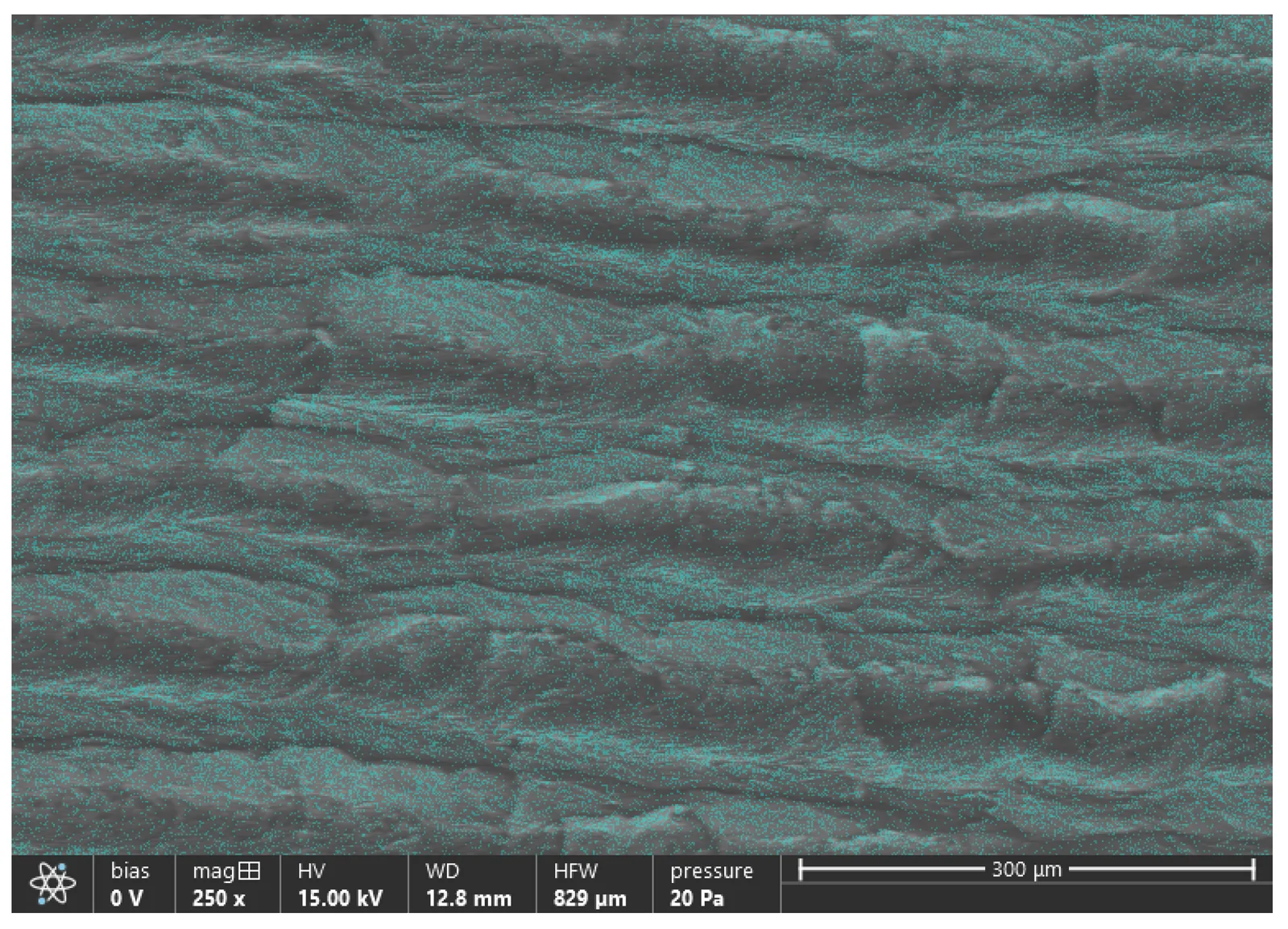
EDS mapping of Ag in the Ag@LIG sample.

To further examine the distribution and uniformity of Ag across the sample, EDS mapping was performed (Figure 5). The mapping confirms that Ag nanoparticles cover the entire surface of Ag@LIG, albeit with some inhomogeneity related to the line-by-line scanning regime of the laser.

Notably, the silver content determined by XPS (23 at.%) is substantially higher than the EDS values. This discrepancy is rationalised by the difference in the probing depths of the two techniques. XPS is inherently surface-sensitive, with an information depth of approximately 5–10 nm, and thus primarily detects silver nanoparticles present at or very near the outermost surface of the porous graphene network. In contrast, EDS probes a much larger interaction volume (typically on the order of 1–3 μm in depth), providing a bulk-averaged composition that includes the underlying carbon matrix and pore interiors, where silver may be less abundant. Consequently, the XPS result reflects a higher surface concentration of Ag nanoparticles, consistent with their partial exposure at the graphene surface, while the EDS data provide a more conservative estimate of the overall silver loading across the entire sampled volume.

Overall, the combined SEM-EDS, Raman, and XPS results confirm the successful formation of Ag@LIG with embedded metallic silver nanoparticles, a porous graphene architecture, and a surface chemistry favorable for electrocatalytic applications.

### 3.2. Electrocatalytic Activity Toward Oxygen Reduction Reaction

#### 3.2.1. Cyclic Voltammetry

**Figure 6:**
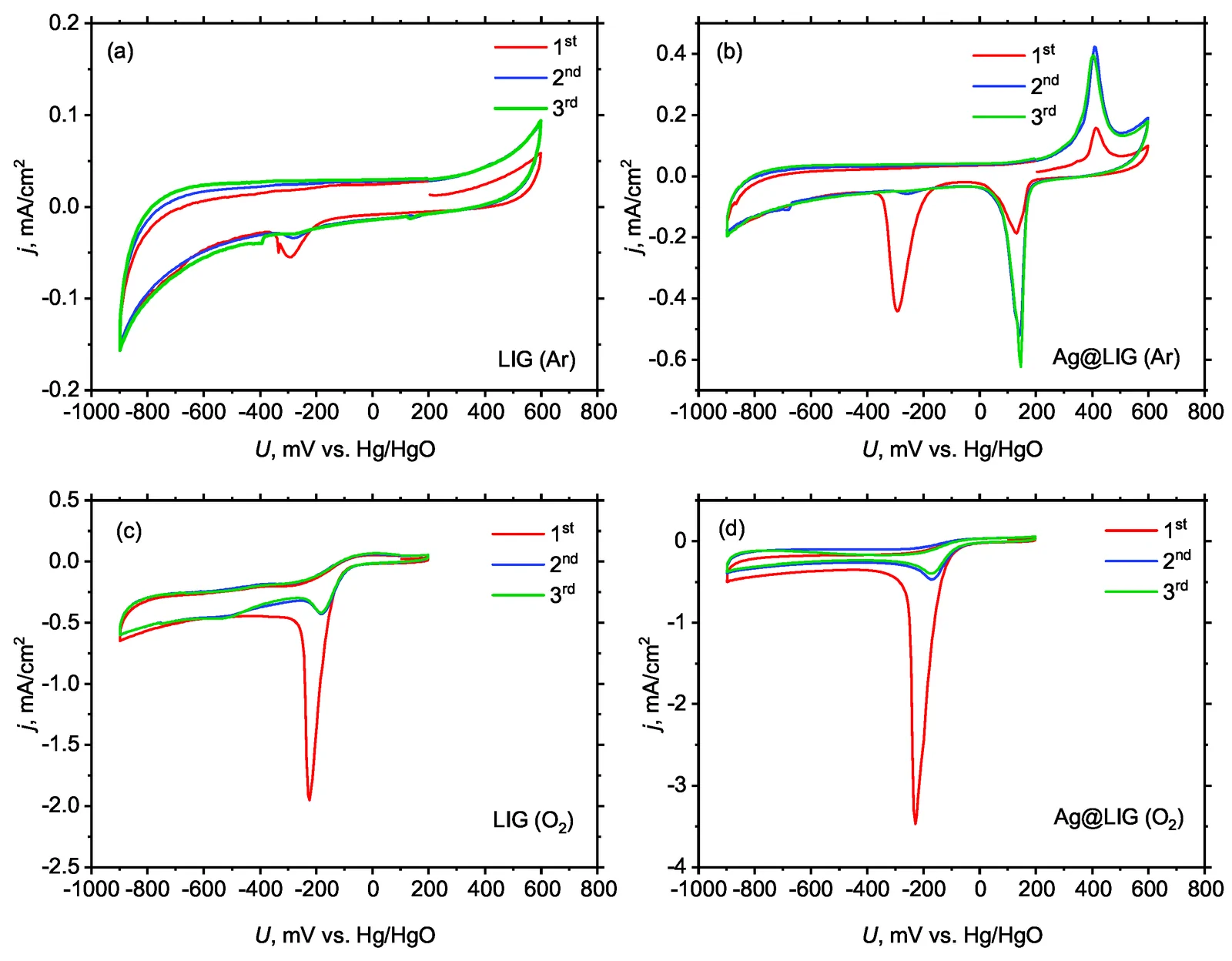
Cyclic voltammetry (first three cycles) for LIG (**a**) and Ag@LIG (**b**) in the argon-saturated electrolyte and for LIG (**c**) and Ag@LIG (**d**) in the oxygen-saturated electrolyte. The current is normalized to the geometric area of the disk. Scan rate is 5 mV/s.

The electrochemical characterization was initiated with cyclic voltammetry (CV) measurements in argon-saturated electrolyte, as shown in Figure 6a,b for LIG and Ag@LIG, respectively. Subsequently, the electrolyte was purged with oxygen, and voltammograms were recorded in the oxygen-saturated electrolyte (Figure 6c,d). In argon-saturated electrolyte, a reduction peak is observed at approximately −0.3 V vs. Hg/HgO during the first cathodic scan. This peak disappears in subsequent cycles, indicating an irreversible electrochemical modification of the carbon material. Similar behavior is often observed during the electroreduction of graphene oxides (GOs) and is typically associated with the reduction of oxygen-containing functional groups, such as carbonyl and epoxy groups, which give reduction peaks in the region of −0.2 to −0.3 V vs. Hg/HgO [66,67,68,69]. However, in our case, XPS analysis reveals that the total oxygen content in the Ag@LIG sample is below 5 at.%, which is insufficient to account for the magnitude of the observed reduction peak. Therefore, we attribute this first-cycle reduction peak to the electroreduction of molecular oxygen that remains adsorbed on the nitrogen-rich sites of the carbon matrix. It has been shown that carbon atoms adjacent to nitrogen atoms act as preferential adsorption sites for O_2_ [70,71,72,73], where oxygen binds strongly enough to remain adsorbed during argon purging. Thus, the reduction peak observed in argon-saturated electrolyte corresponds to the electroreduction of this pre-adsorbed oxygen. After subsequent purging with oxygen, the same peak reappears with significantly higher intensity, which is attributed to the higher concentration of dissolved O_2_ and enhanced adsorption on nitrogen sites.

For Ag@LIG in argon-saturated electrolyte, additional oxidation and reduction peaks are observed, which are associated with the redox behavior of silver (Figure 6b) [74,75,76]. The anodic peak at approximately +0.4 V is attributed to the formation of Ag_2_O: 2Ag + 2OH^−^→ Ag_2_O + H_2_O + 2e^−^, while the cathodic peak at approximately +0.15 V corresponds to the reverse reduction of Ag_2_O to metallic silver [74]. To avoid silver oxidation and reduction, the anodic potential limit was reduced to +0.2 V in experiments with oxygen-saturated electrolytes (Figure 6c,d). In the presence of oxygen, a pronounced reduction peak is observed during the first cathodic scan for both LIG and Ag@LIG (Figure 6c,d), which is significantly attenuated in subsequent cycles. From the second cycle onward, the voltammograms stabilize, and the peak at approximately −0.2 V is attributed to the ORR.

The substantial decrease in the ORR peak current in subsequent cycles is likely due to the irreversible deactivation of nitrogen active sites by the oxidative attack of ORR intermediates [77]. Several mechanisms have been proposed to explain this deactivation. First, pyridinic and pyrrolic nitrogen species can undergo oxidative dissolution by highly reactive oxygen intermediates generated during the ORR, forming ${\mathrm{NO}}_{3}^{-}$ and ${\mathrm{NO}}_{2}^{-}$ ions that leach into the electrolyte [78]. Second, the carbon matrix itself may corrode in regions adjacent to nitrogen dopants, particularly under the influence of peroxide species, leading to the loss of active site integrity [79]. Third, active nitrogen species may be converted into less active N–C–O functionalities through oxidation, a process that is thermodynamically favorable under the applied potential [73,80]. Although these oxidized species may retain some catalytic activity, they are significantly less active than the pristine nitrogen centers, which explains the observed attenuation of the reduction peak after the first cycle [73]. This interpretation is consistent with the behavior observed for polymer-derived nitrogen-doped carbons, where the presence of pyrrolic nitrogen at the edges of the carbon network leads to irreversible electrochemical oxidation and loss of catalytic activity under operating potentials [81]. Collectively, these mechanisms account for the rapid deactivation of nitrogen centers and the substantial decrease in peak current after the first cycle.

#### 3.2.2. Rotating Ring-Disk Electrode Measurements

**Figure 7:**
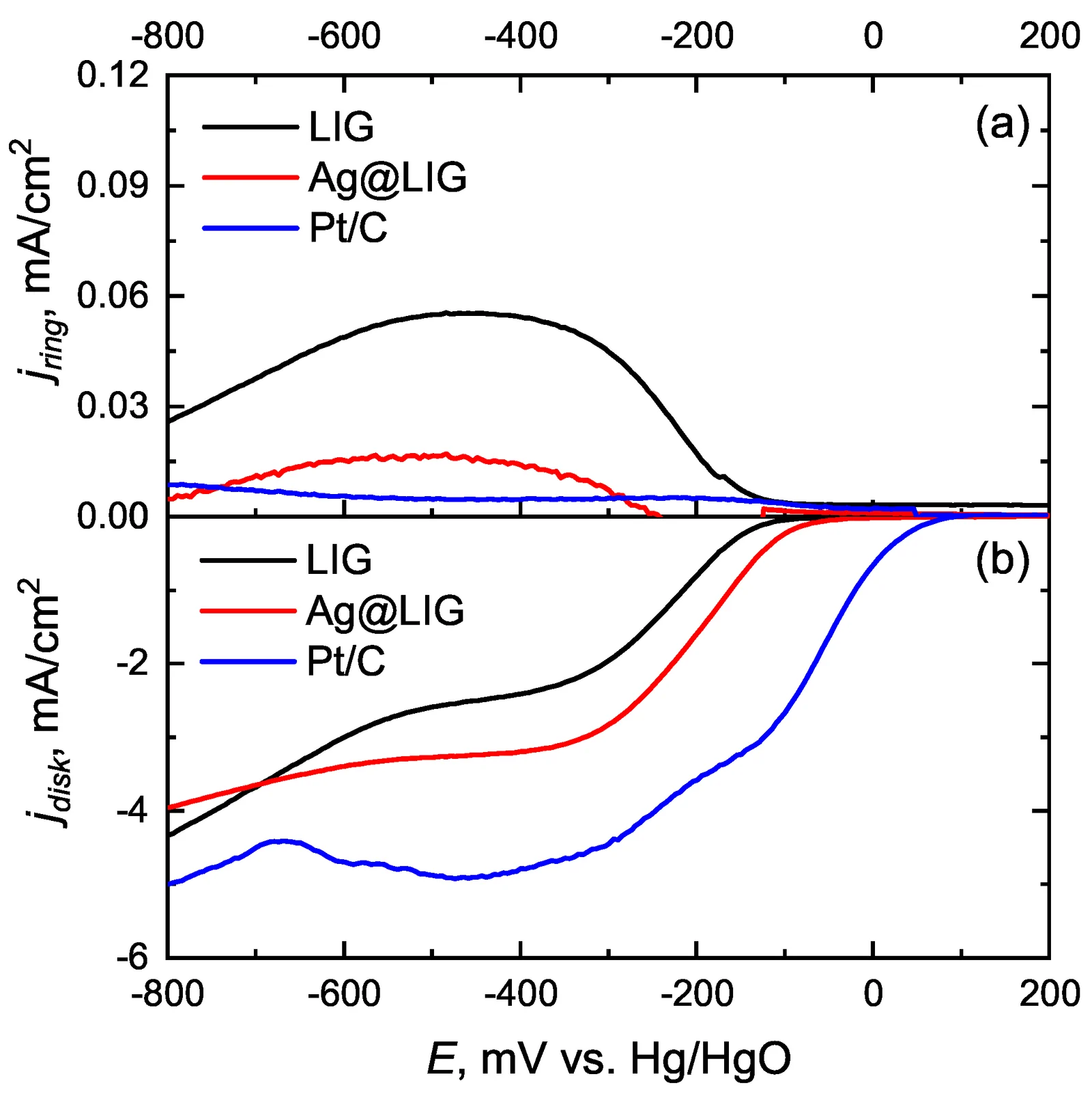
RRDE measurements of ORR activity (scan rate: 5 mV/s; rotating rate: 1500 rpm); (**a**) ring current normalized to the geometric area of the ring; (**b**) disk current normalized to the geometric area of the disk.

After CV measurements in the O_2_-saturated electrolyte, the same samples were used to study the electrocatalytic activity using the Rotating Ring-Disk Electrode (RRDE) technique. Figure 7b shows the ORR polarization curves for LIG, Ag@LIG, and a commercial Pt/C catalyst. To obtain the pure faradaic ORR current, the capacitive current (measured in Ar-saturated electrolyte) was subtracted from the disk current. Figure 7a shows the ring current associated with the oxidation of ${\mathrm{HO}}_{2}^{-}$ ions formed on the disk via the 2e^−^ pathway of the ORR in alkaline medium. The number of electrons transferred per oxygen molecule (*n*) was calculated using the following equation:

(1) $$n=4\frac{I_{disk}}{I_{disk}+I_{ring}/Q}$$

where $I_{disk}$ and $I_{ring}$ are the disk and ring currents, respectively, and *Q* is the collection efficiency of the RRDE tip (*Q* = 0.267), which was obtained using the ${\mathrm{Fe}(\mathrm{CN})}_{6}^{3-/4-}$ redox couple and gave a value close to the passport value (0.256).

The peroxide percentage (% ${\mathrm{OOH}}^{-}$ in alkaline media) was calculated using the following equation:

(2) $$\%OOH^{-}=200\frac{I_{ring}/Q}{I_{disk}+I_{ring}/Q}$$

**Figure 8:**
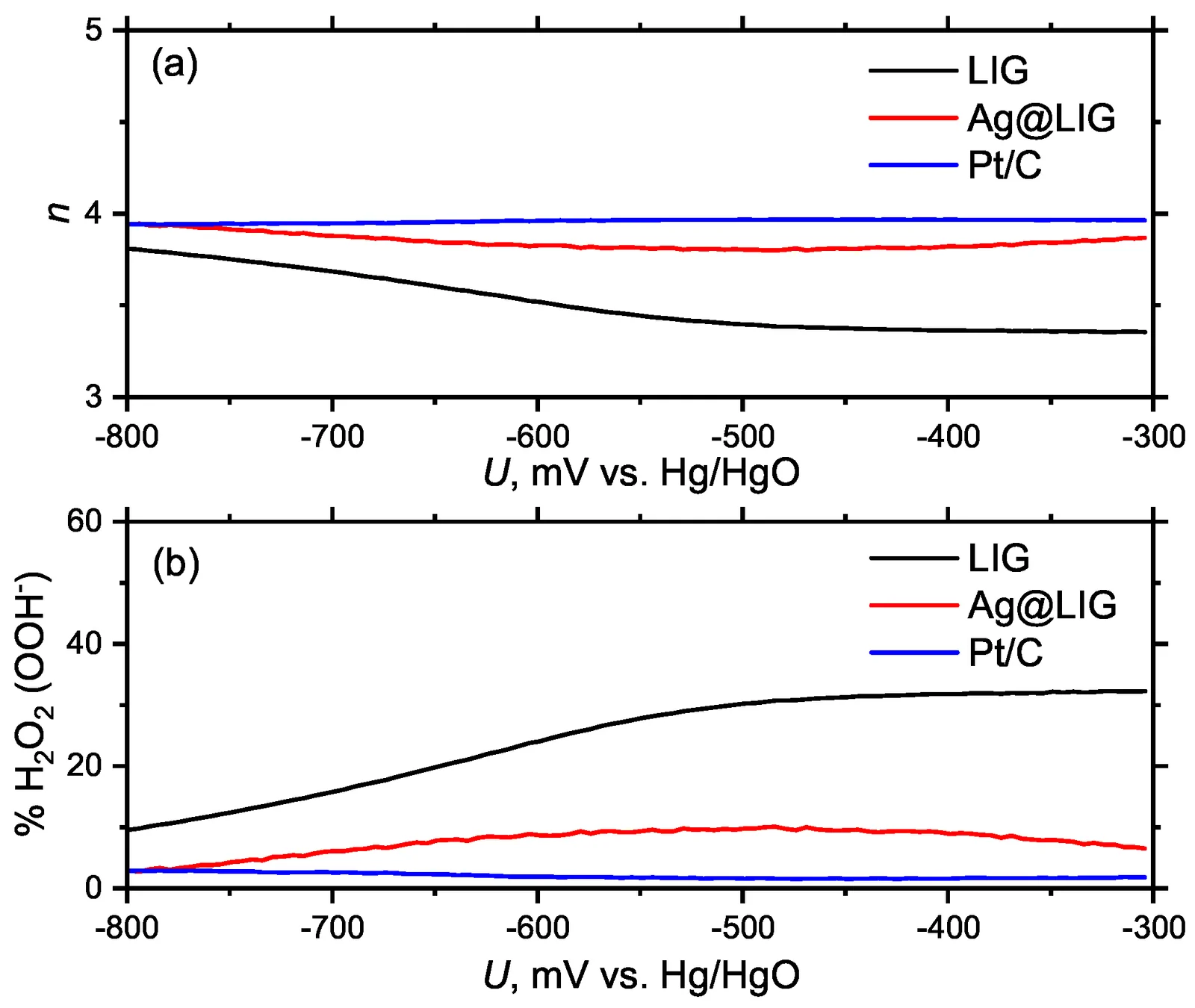
(**a**) Number of transferred electrons depending on the disk potential; (**b**) Percentage of peroxide production along ORR curves.

The polarization curve for LIG exhibits two distinct waves (Figure 7b), a feature often observed for metal-free carbon catalysts [82,83,84] and attributed to a (2 + 2)$e^{-}$ reaction mechanism [84]. In the potential range of the first plateau (approximately −0.5 to −0.3 V vs. Hg/HgO), the ring current is high, indicating a substantial yield of ${\mathrm{HO}}_{2}^{-}$. The calculated electron transfer number in this region is 3.3–3.4 (Figure 8a), corresponding to ∼ 30% of peroxide yield. At more negative potentials, ${\mathrm{HO}}_{2}^{-}$ ions trapped within the porous catalyst layer undergo further reduction, and *n* approaches 4 [84,85,86].

For Ag@LIG, the ORR onset potential is approximately 30 mV more positive than that of LIG (Figure 7b) and is equal to ∼−0.09 V vs. Hg/HgO, which corresponds to ∼0.78 V vs. RHE; this value is in agreement with measurements for single-crystal silver [87] and silver-based electrocatalysts on carbon supports [8]. Moreover, the ring current is significantly lower (Figure 7a), and the electron transfer number is close to 4 over the entire potential range, comparable to the commercial Pt/C catalyst (Figure 8a). Peroxide percentage is less than 10% within the whole voltage range (Figure 8b). Thus, the incorporation of metallic silver nanoparticles significantly enhances the catalytic efficiency of LIG, shifting the ORR pathway toward the desired 4$e^{-}$ reduction of $O_{2}$ to ${\mathrm{OH}}^{-}$.

To assess reproducibility, RRDE measurements for LIG and Ag@LIG were repeated with three independently prepared electrodes. The key parameters are summarized in Table 6 with mean values and standard deviations and compared with the reported Ag/C catalysts in the literature.

#### 3.2.3. Stability Tests

**Figure 9:**
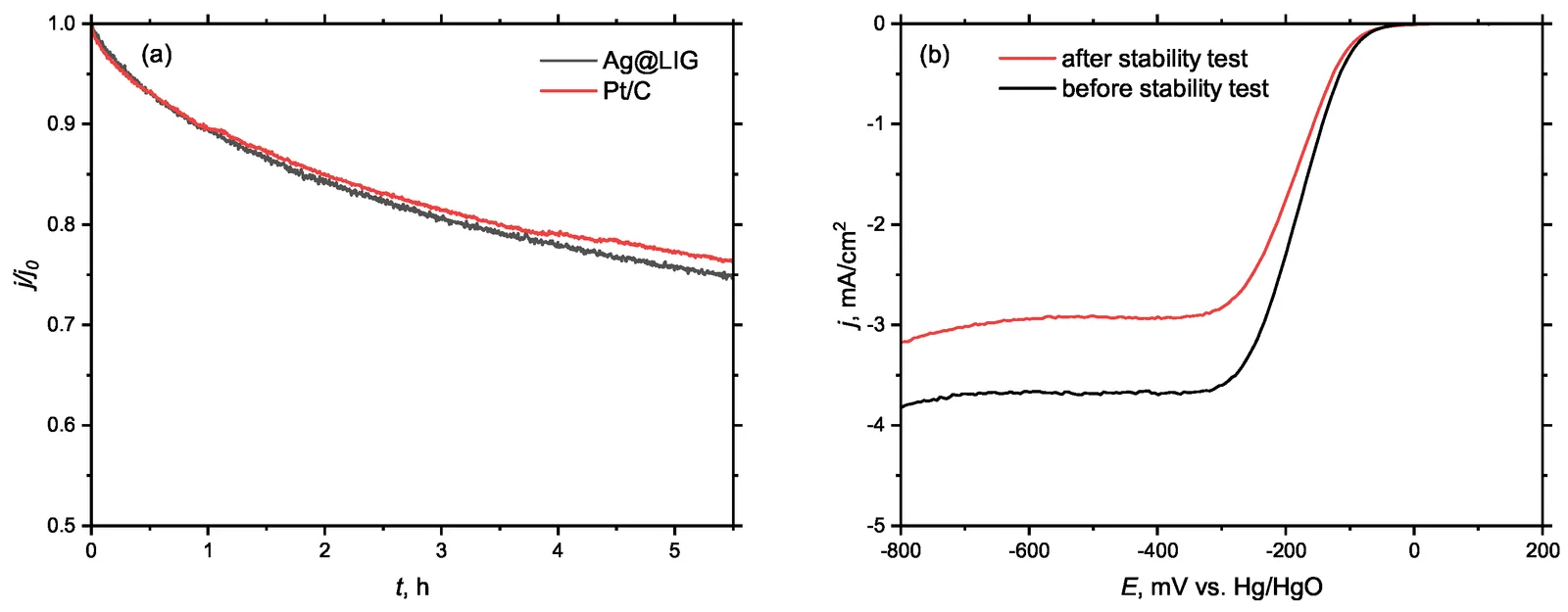
(**a**) Chronoamperometry at −0.4 V vs. Hg/HgO for Pt/C and Ag@LIG catalysts; (**b**) ORR polarization curves for Ag@LIG at 1500 rpm before and after the stability test.

The stability of the Ag@LIG catalyst was evaluated by chronoamperometry at a fixed potential of −0.4 V vs. Hg/HgO for 5.5 h in $O_{2}$-saturated 0.1 M KOH under continuous rotation at 1000 rpm, and compared with commercial Pt/C under identical conditions (see Figure 9a). Ag@LIG retained a substantial fraction of its initial current, exhibiting stability comparable to that of commercial Pt/C. Both catalysts showed a ∼25% current loss over 5 h, which is typical for Pt/C catalysts and consistent with the literature [92,93]. Figure 9b shows ORR polarization curves for Ag@LIG recorded before and after the stability test. A decrease in the limiting current observed after the test agrees with the current loss observed in chronoamperometry.

## 4. Conclusions

In this study, we successfully synthesized silver nanoparticle-decorated laser-induced graphene (Ag@LIG) using a simple, solid-state, reagent-free, and one-step laser direct writing approach from a polyimide film coated with an edible silver leaf. The laser treatment simultaneously converted the polymer into a porous three-dimensional graphene network and incorporated metallic silver nanoparticles derived from the metal leaf into the carbon matrix.

SEM and TEM analyses revealed that the resulting Ag@LIG retains the characteristic porous morphology of LIG, while silver nanoparticles with sizes ranging from 5 to 50 nm are embedded within the graphene sheets. XPS analysis showed a silver content of 24 at.% at the surface, while EDS measurements yielded lower values of 1.6–7.3 at.%, reflecting the surface-sensitive nature of XPS and the bulk-averaged probing depth of EDS. The silver nanoparticles are predominantly metallic, as confirmed by the Ag ${3d}_{5/2}$ binding energy of 368.2 eV. XPS revealed a higher nitrogen content in Ag@LIG than in LIG, with pyrrolic nitrogen as the dominant configuration. However, the role of nitrogen in the enhanced ORR performance should be considered cautiously, as additional control experiments would be required to separate its contribution from that of the Ag nanoparticles. Raman spectroscopy showed an increase in the $I_{D}$/$I_{G}$ ratio from 0.86 to 1.24 upon silver incorporation, indicating a rise in structural disorder consistent with the incorporation of nitrogen and silver nanoparticles into the graphene lattice.

Electrochemical characterization demonstrated that the incorporation of silver nanoparticles significantly improves the ORR performance of LIG. The ORR onset potential shifts by approximately 30 mV toward more positive values, reaching approximately −0.09 V vs. Hg/HgO. RRDE measurements revealed that Ag@LIG promotes a predominantly four-electron pathway (*n* ≈ 4 over the entire potential range) with substantially lower ring current compared to pristine LIG, indicating a reduced peroxide yield. In contrast, pristine LIG exhibits a (2 + 2)$e^{-}$ pathway with *n* = 3.3–3.4 in the first wave and significant ${\mathrm{HO}}_{2}^{-}$ formation. The enhanced performance is associated with the Ag@LIG composite, in which both metallic Ag nanoparticles and the N-doped carbon matrix may contribute. The nitrogen-rich carbon matrix, predominantly in the form of pyrrolic nitrogen, is known to provide favorable sites for $O_{2}$ adsorption, while the embedded Ag nanoparticles are known to facilitate O–O bond cleavage. However, additional control experiments—such as Ag-free N-doped LIG, Ag on undoped carbon, and systematic variation of Ag loading—would be required to fully separate the contributions of each component.

Overall, the solid-state laser synthesis approach offers a straightforward and scalable route for fabricating Ag-carbon hybrid electrocatalysts, avoiding the use of solvents, reducing agents, and complex preparatory steps. The Ag@LIG composite demonstrates enhanced ORR activity, positioning it as a promising alternative to platinum-based catalysts for alkaline fuel cell applications.

## Figures and Tables

**Table 1:** Catalyst loadings.

Catalyst	Total Loading (mg/cm^2^)	Carbon Loading (mg/cm^2^)	Metal Loading (mg/cm^2^)
LIG	0.73	0.73	—
Ag@LIG	0.73	0.43–0.64	0.09–0.30 (Ag)
Pt/C	1.22	0.73	0.49 (Pt)

**Table 2:** Raman peaks of LIG and Ag@LIG samples.

Sample	Band	Position (cm^−1^)	Peak Height (a.u.)	FWHM (cm^−1^)	Peak Area (a.u.)
LIG	D	1337.20 (±0.43)	2193	54	155,992
	G	1570.45 (±0.30)	2545	43	141,352
	$D^{\prime }$	1609.58 (±1.20)	366	25	9821
	2D	2677.40 (±2.58)	489	72	45,398
Ag@LIG	D	1345.67 (±0.13)	6784	49	428,049
	G	1577.63 (±0.13)	5469	42	27,7210
	$D^{\prime }$	1613.70 (±0.20)	2157	25	57,192
	2D	2684.86 (±1.19)	824	56	49,113

**Table 3:** Binding energies (BEs) and relative atomic concentrations of elements derived from high-resolution XPS spectra of the LIG sample.

Component	BE, eV (CS)	Atomic %
**C 1s**		
sp^2^	284.29	76.26
C–O, C–N	285.78 (+1.49)	7.60
C=O	287.24 (+2.95)	3.75
–COO	288.67 (+4.39)	2.26
π–π*	290.56 (+6.48)	–
		**(89.87)**
**N 1s**		
pyrrolic	399.76	1.78
graphitic	401.84	0.17
–NO	403.70	0.07
–NO_2_	405.80	0.16
		**(2.18)**
**O 1s**		
C=O	531.75	3.84
C–O	533.07	3.63
		**(7.95)**

CS = chemical shift relative to the main C 1s sp^2^ component at 284.29 eV. Total atomic percentages are shown in bold.

**Table 4:** Binding energies (BEs) and relative atomic concentrations of elements derived from high-resolution XPS spectra of the Ag@LIG sample.

Component	BE, eV (CS)	Atomic %
**Ag 3d**		
Ag metal	368.20	23.99
		**(23.99)**
**C 1s**		
sp^2^	284.18	39.43
C–O, C–N	284.95 (+0.77)	14.40
C–O, C–N	285.91 (+1.73)	4.22
C=O	287.82 (+3.64)	2.25
π–π*	290.99 (+6.81)	–
		**(60.30)**
**N 1s**		
pyrrolic	399.49	10.15
graphitic	401.16	0.68
		**(10.83)**
**O 1s**		
C=O, Ag–O–C	531.80	2.95
C–O	533.64	1.93
		**(4.88)**

CS = chemical shift relative to the main C 1s sp^2^ component at 284.18 eV. Total atomic percentages are shown in bold.

**Table 5:** Element composition of Ag@LIG based on EDS measurements.

Element	Area 1. Atomic %	Area 2. Atomic %
C	89.7	98.4
O	3.0	–
Ag	7.3	1.6

**Table 6:** Comparison of ORR performance of Ag@LIG and LIG with the reported Ag/C catalysts.

Catalyst	Onset Potential at 0.1 mA/cm^2^ (mV vs. Hg/HgO) *	Electron Tranfer Number *n*	Current Density at −0.6 V vs. Hg/HgO (mA/cm^2^)	Ref.
LIG	−119 ± 0	3.27 ± 0.20	3.03 ± 0.10	This work
Ag@LIG	−89 ± 13	3.72 ± 0.10	3.1 ± 0.39	This work
Ag/Vulcan	−211 **	2.1	∼2	[88]
Ag/Vulcan	−278 **	3.5	∼3.5	[89]
10 wt.% Ag/C	—	2.7	∼2.5	[90]
10 wt.% Ag/rGO	−65	2.9	∼3	[91]
40 wt.% Ag/rGO	−5	3.8	∼8	[91]

* Literature potentials reported vs. RHE were converted to the Hg/HgO (1 M KOH) scale using $E_{\mathrm{Hg}/\mathrm{HgO}}=E_{\mathrm{RHE}}-0.059\times \mathrm{pH}-0.098$ V, where pH corresponds to the electrolyte used in the original study. ** In these references, the authors did not specify the current density criterion used to determine the onset potential, nor did they present log-scale current plots; the values are given as reported.

## Data Availability

The raw data supporting the conclusions of this article will be made available by the authors on request.

## Abbreviations

The following abbreviations are used in this manuscript:

AEMFC	anion exchange membrane fuel cell
Ag@LIG	silver nanoparticle-decorated laser-induced graphene
AgNP	silver nanoparticle
BE	binding energy
CS	chemical shift
CV	cyclic voltammetry
EDS	energy-dispersive X-ray spectroscopy
GO	graphene oxide
LIG	laser-induced graphene
LSV	linear sweep voltammetry
ORR	oxygen reduction reaction
PEMFC	proton exchange membrane fuel cell
PI	polyimide
RDE	rotating disk electrode
RRDE	rotating ring-disk electrode
RSF	relative sensitivity factor
SEM	scanning electron microscopy
SERS	surface-enhanced Raman scattering
TEM	transmission electron microscopy
XPS	X-ray photoelectron spectroscopy
